# High School Teachers’ Experiences of Consumer Technologies for Stress Management During the COVID-19 Pandemic: Qualitative Study

**DOI:** 10.2196/50460

**Published:** 2023-11-15

**Authors:** Julia B Manning, Ann Blandford, Julian Edbrooke-Childs

**Affiliations:** 1 UCL Interaction Centre Department of Computer Science University College London London United Kingdom; 2 UCL Institute of Healthcare Engineering University College London London United Kingdom; 3 Evidence-based Practice Unit University College London and Anna Freud Centre London United Kingdom

**Keywords:** teachers, stress, self-management, digital health, technology, qualitative, secondary schools, high schools, wearables, context

## Abstract

**Background:**

Stress in education is an adverse reaction that teachers have to excessive pressures or other types of demands placed on them. Consumer digital technologies are already being used by teachers for stress management, albeit not in a systematic way. Understanding teachers’ experiences and the long-term use of technologies to support stress self-management in the educational context is essential for meaningful insight into the value, opportunity, and benefits of use.

**Objective:**

The aim of this study was first to understand teachers’ experiences of consumer technologies for stress management. They were chosen by teachers from a taxonomy tailored to their stress management. The second aim was to explore whether their experiences of use evolved over time as teachers transitioned from working at home during lockdown to working full time on school premises.

**Methods:**

A longitudinal study intended for 6 weeks in the summer term (2020) was extended because of COVID-19 into the autumn term, lasting up to 27 weeks. Teachers chose to use a Withings smartwatch or the Wysa, Daylio, or Teacher Tapp apps. In total, 2 semistructured interviews and web-based surveys were conducted with 8 teachers in South London in the summer term, and 6 (75%) of them took part in a third interview in the autumn term. The interviews were analyzed by creating case studies and conducting cross-case analysis.

**Results:**

The teachers described that the data captured or shared by the technology powerfully illustrated the physical and psychosocial toll of their work. This insight gave teachers permission to destress and self-care. The social-emotional confidence generated also led to empathy toward colleagues, and a virtuous cycle of knowledge, self-compassion, permission, and stress management action was demonstrated. Although the COVID-19 pandemic added a new source of stress, it also meant that teachers’ stress management experiences could be contrasted between working from home and then back in school. More intentional self-care was demonstrated when back in school, sometimes without the need to refer to the data or technology.

**Conclusions:**

The findings of this study demonstrate that taking a situated approach to understand the real-world, existential significance and value of data generates contextually informed insights. Where a strategic personal choice of consumer technology is enabled for high school heads of year, the data generated are perceived as holistic, with personal and professional salience, and are motivational in the educational context. Technology adoption was aided by the pandemic conditions of home working, and this flexibility would otherwise need workplace facilitation. These findings add to the value proposition of technologies for individual stress management and workforce health outcomes pertinent to educators, policy makers, and designers.

## Introduction

### Background

Workplace psychological stress continues to be a pervasive problem for many. When employees talk about stress, they are usually alluding to negative “distress,” which is an unwanted and possibly damaging burden, rather than positive “eustress,” a motivator for growth, development, and change [1,2]. Psychological stress continues to be endemic among teachers. Before the pandemic, teachers recorded the highest employee average for stress, anxiety, and depression with 2170 cases per 100,000 educators based on National Health Service general practitioner data on working days lost [3]. The socioeconomic effects extend beyond work absence as stress is a known risk factor for both psychological conditions such as depression and physiological diseases such as cardiovascular disease and diabetes [2,4-6]. There is an organizational responsibility for educational leadership to reduce the stressors experienced by teaching staff. However, teachers knowing individually how they can better manage their stress is crucial for well-being and to avoid burnout, especially high school teachers with pastoral responsibilities in city schools, who report greater levels of pressure and strategic time management than colleagues in other roles or locations [7-10].

Understanding the value of digital support for teachers’ stress management requires knowledge of their experiences in the workplace, particularly with consumer technologies that are easily available. Of the limited teacher studies on digitally supported stress management, few focus on stress alone but do indicate that personal insight is generated [11-13]. Some research emphasizes burnout prevention, which is a more severe occupational outcome of ongoing stress [14]; others explore disorders such as depression or insomnia associated with stress and report on stress measures [15,16]. Technology has also been deployed as a small component of a wider stress intervention [17], or teachers are included in a wider cohort of participants [18]. We know that teachers use available consumer digital tools to support their stress management even if they find that doing so is constrained by educational contexts such as physical space, social relationships, and school culture [19]. Although some studies with teachers have used consumer wearables [12,13], knowledge of teachers’ actual experiences with and consequences of digitally supported stress management and what is meaningful to them in their occupational context is sparse. Such knowledge could inform both technology choice and personal understanding of the response to pressure, thus improving stress management strategies.

Therefore, the aims of this study were to address this gap by giving teachers an informed choice of technology to support their stress management and explore whether and how the technology used in the context of their work was valuable and significant for them. Unexpectedly, this study got underway just when the COVID-19 pandemic hit. This meant that we had the exceptional opportunity to explore teachers’ experiences both when working from home and then when back on the school premises, although the latter were still subject to hygiene restrictions. Inevitably, the pandemic added another source of stress, yet self-report survey data imply that COVID-19 did not change the prevalence of stress overall or the willingness of staff to report it. In both 2019 and 2021, a total of 84% of senior school leaders reported stress, and 60% and 57% of staff, respectively, said that they would not share an unmanageable stress issue with their employer [20,21].

In this paper, in common with much of the literature, we position stress as normal (not a disease) and experienced by most teachers to avoid, as much as possible, any sense of fault or stigma given that research shows that this is still an issue [22,23]. The UK National Institute for Health and Care Excellence definition of stress can be adapted to an educator-focused description, viz, “the adverse reaction *teachers* (people) have to excessive pressures or other types of demand placed on them” [24].

The World Health Organization positions stress as a determinant of mental health, which it defines as “a state of mental well-being that enables people to cope with the stresses of life, to realize their abilities, to learn well and work well, and to contribute to their communities” [25]. For this study, stress is considered in the context of education, related to both mental health and physical well-being, with recognized symptoms in teachers [26-28]. This aligns with other teaching and computing literature where stress is described as an experience or reaction of discomfort [29], strain [30], and tension [31] and positioned as a workplace problem [32]. Given the variability with which the term “context” can be used and the difficulty deciphering which context is being referred to, we also drew on multidisciplinary domains of education, human factor, and informatics literature [33-37] to define the education workplace as the macrocontext; the temporal, physical, social, and cultural factors as mesocontexts; and the individual and their personally chosen strategy as the microcontext.

In this paper, we review work on understanding the effectiveness of digital stress interventions for teachers and in other workplaces and what is known about employees’ experiences with some of the interventions that have been studied. We then describe the digitally supported mechanisms that were offered to teachers to support their stress management strategies. We then present our study and results and discuss the significance and value of data and technologies to teachers and how this experience supported a more holistic understanding of stress and the need for self-care.

### Related Work

#### Technologies in the Workplace Context

Previous studies on digital stress interventions with teachers have tended to focus on treatment outcomes and not on experiences with the intervention or with consumer technologies. Internet-based cognitive behavioral therapy (iCBT), previously evidenced to reduce depression, was very effective in improving teachers’ stress-associated insomnia [16,38]. Tailored third-wave iCBT (internet-based problem-solving therapy) showed a medium-sized but long-term effect on teachers’ stress [15]. Third-wave iCBT and positive psychology had a sustained, medium to large effect on stress reduction in a group including teachers in management [18]. Although there was no longer-term follow-up, a 4-week third-wave iCBT course reduced burnout factors in mostly younger teachers and had a retention rate of 92.8% [14]. Notably, the design allowed for flexibility in both chosen strategies and scheduling. Studies mostly agree that guided or face-to-face interventions are more efficacious, but many also point out the barriers of time, cost, stigma, and provider capacity preventing such interventions from being a choice for many.

Just a few studies have provided insights into teachers’ experiences with technology. One iCBT and another wearable study reported enabling personal insights [12-14]. Together with another study, it was shown that awareness of stress was created through personal data, psychoeducation, or colleagues’ experiences and that autonomy in strategy choice, timing, or use of time was valued [12,14,39]. Studies have also reported appreciating mutual social support, connectedness on the internet and anonymity [39], or informational support via an app [17]. Wearable data have been shown to motivate more physical activity (unrelated to stress) [40], and an understanding of in- and out-of-work stressors was generated through a teacher-tailored web-based program [15]. On the negative side, not being able to translate knowledge of stress from collated wearable data into action has been reported [12]. Only the studies using wearables seem to have used consumer technology rather than purpose-built programs.

Other workplace digital stress intervention studies are often office based in technology companies, so experiences with them may not be as relevant to teachers because of the constrained nature of teachers’ schedules. However, findings have confirmed that digital tools can offer autonomy through prescheduled or just-in-time stress management solutions [32,41]. In addition, flexibility over time and place of access, reminders, and shorter intervention duration are appreciated [42], which could appeal to the time-pressured education sector. Choice over manual and automated personalization of both content and delivery schedule were valued by desk workers [41] and could be enhanced by system feedback on the efficacy of chosen interventions or on causal links [43]. Although not assessed in or for the work environment, personalization within stress management apps is the most frequently implemented strategy [44].

#### How Technologies Support Stress Management

Experiences with technology are themselves enabled by the elements and characteristics of features or mechanisms [45]. These features have been described in a simplified conceptualization as reflection, treatment, social support, or entertainment for stress self-management based on the mechanism or rationale for interaction [46]. There is some research on how these features have been deployed to help with the stress management of employees that relates to the experiences that technologies can offer.

The purpose of reflection is to gain self-knowledge by exploring relevant data, and it is recognized as the fourth stage in the personal informatics (PI) model, where the data are self-generated [47]. Reflection can occur during data collection [48], also termed reflection-in-action, or after the event, reflection-on-action [49,50]. Digital stress tracking is still new, and experiences of its use in the workplace are few, although studies recognize the importance of contextualized investigation [51]. Generating awareness of stress was valued among IT workers [52,53], but a study of working-age adults already using consumer wearables reported that most did not realize that they had features to track and illustrate stress [54]. Visualizations of information have been shown to generate powerful affective ties between people and their data [12,55], but where consumer technology had not been acquired for the purpose of stress management, the lack of real-time measures or alerts resulted in reduced engagement [54]. Reflection is not limited to PI; it can also be facilitated through apps supporting emotional self-awareness [56,57], chatbots offering guided therapy [41,58], or timely reminders [32,41,59].

In addition to reflection, digital tools can be a medium for practical support and therapy. Treatment and self-management for stress can include iCBT, third-wave iCBT, mindfulness, web-based forums, psychoeducation, and internet stress management interventions that can include any of the aforementioned. Exploring employees’ experiences from interaction with such treatments is limited but has included determinants of adherence. Employees were more likely to stick with a tailored stress intervention when given either content- or adherence-focused guidance and support [60], and reviews tend to agree that employees prefer and do better when given practical support with digital interventions [61-63]. For a nonclinical adult population, humor, relevance, encouraging content, and flexibility were meaningful for the sustained use of a positive psychology audiovisual program via an app or website [64]. Reflection and treatment can be brought together, as demonstrated when machine learning was applied to tracking data, leading to suggested interventions for stress-mitigating planning and action by office workers [53].

A third way of supporting stress management is through peer and social support. This is powerful as mutual understanding creates solidarity and empathy, and communities that have shared experiences support individual meaning making [65]. Teachers described this from using a web-based forum in Hong Kong [39], and sharing ideas for stress management was valued by students [66]. In addition, IT workers found that social networks leveraged humor and intimacy, which could reduce individual perceived stress levels [67]. Replicating empathy using artificial agents (chatbots) has been challenging [68,69] even when designed for stress in a specific context [70] with much research to come [71]. However, empathy has been demonstrated in a public cohort reporting depression [58], and ChatGPT responses to various patient questions were perceived by health professionals as more empathetic than physician responses (although not clinically validated) [72].

Finally, the rationale for games is that they can help with stress through positive psychology, relaxation, improved self-perception, or psychoeducation [67,73-75]. Signposting through contextually informed machine learning to popular web applications that support therapeutic strategies, including fun and games, has demonstrated increased self-awareness of stress and new ways to deal with it [76]. However, app designers do not yet seem to be exploiting the potential of gamification techniques [75,77-79]. Studies have shown that stress reduction is possible as a by-product of digital technology use, as in IT employees using gamification to build resilience [57] or with nurses using an exercise video and Nintendo Wii [80].

Thus, we have some insights into how technology design has supported stress management but little knowledge of how or why technologies could help teachers, teachers’ experiences of use, and what was valuable or significant to them.

### This Study’s Aims

Knowing that there are consumer technologies that offer these mechanisms or features that could potentially support stress management strategies, this study focused on high school heads of year (generally referred to in this paper as teachers) using their chosen technology in the context of their work. Our research questions (RQs) were as follows: (1) In the context of their work, how do secondary school heads of year experience their chosen technology for stress self-management? and (2) Does digitally supported stress self-management change from working from home to working back in school?

We took a constructivist approach, embracing subjectivity in the interpretation of the data, a paradigm familiar to both human-computer interaction and education, thus enabling epistemic consistency. The consumer technologies chosen by teachers were the Withings Steel HR smartwatch; Wysa, an artificial intelligence chatbot based on cognitive behavioral therapy techniques; Daylio, a mood diary and activity-tracking app; and Teacher Tapp, a daily teacher survey with an educational blog. The findings complement the identification of facilitators of easy interaction and data presentation and barriers of hidden features and physical, social, and cultural contextual constraints to technology use in the educational context that is under review elsewhere [81].

## Methods

### Overview

The goal of this study was to understand teachers’ experiences of using their chosen consumer technology for stress management. We planned a longitudinal study that used semistructured interviews along with open- and closed-question surveys, feedback questionnaires, and memos as data sources. There are 2 papers on these data; this paper focuses on teachers’ long-term experiences with the technology, and the other focuses on barriers to and facilitators of its use.

### Teacher Recruitment

All participants were recruited from schools in a Multi-Academy Trust based in South London. Owing to the COVID-19 pandemic precipitating a national lockdown, invitations were sent by email following a verbal invitation from the principals, with participation facilitated on the internet. A total of 16 heads of year were invited, and 8 (50%) were still able to participate despite the pandemic, with the inclusion criteria specifying that they should not currently be receiving treatment for a clinical stress disorder such as posttraumatic stress disorder and that a willingness to talk about stress and trial some consumer technology to support their stress management strategy was required. Through the similarity of schools, locations, and roles of the participants (ie, heads of year with pastoral management roles), the planned exploration and comparison of teachers’ experiences with technology through deep, rich, thick narratives and analysis should be more plausible [82].

### Ethical Considerations

This research was approved by the University College London Research Ethics Committee (approval ID: UCLIC_1920_004_Staff_BlandfordManning). The information and consent forms detailed the study protocol, that the data would contribute to a study being undertaken as part of a PhD, and that all data would be stored pseudonymously and securely and would be anonymous once it had been aggregated. In addition, an ethical approach was maintained by reminding participants at every point of contact that they could withdraw from the study and that emotional support was available via the Education Support Partnership. Participants had their choice of technology paid for up to a maximum of £150 (US $183.10).

### Workshops

**Figure 1:**
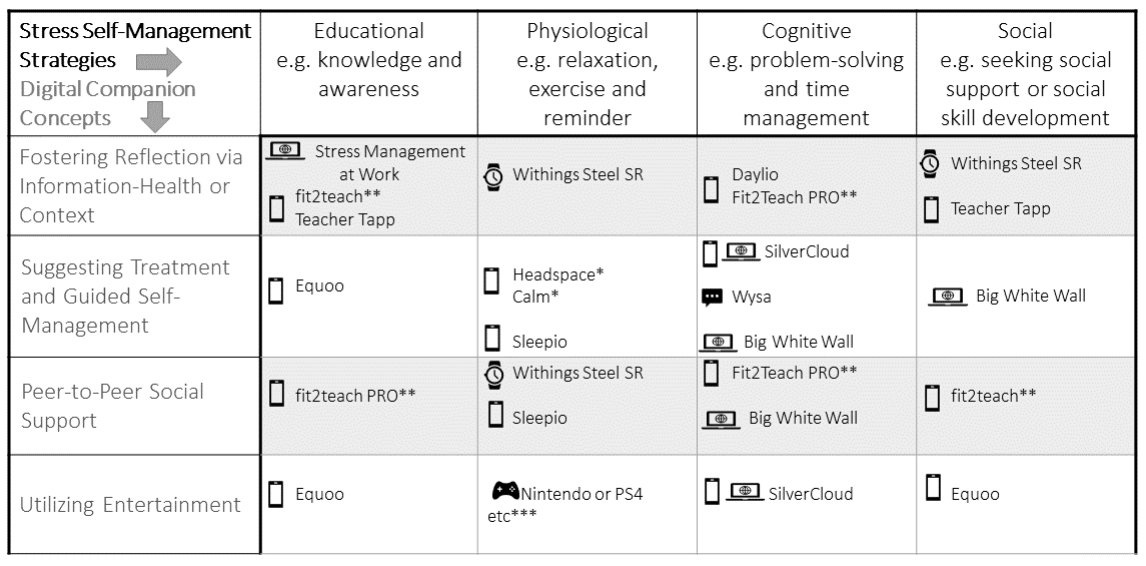
Taxonomy of consumer technologies for stress management presented to the teachers in the workshop. *Only partial encryption of data, **withdrawn due to lack of updates, and ***user to provide own device.

Before the first interview, workshops were conducted to help teachers make an informed choice of consumer technology based on their understanding of their personal stress management strategy and technology preferences. These were held on the web via Zoom (Zoom Video Communications) and used the Mentimeter app for anonymous interaction and Microsoft PowerPoint (Microsoft Corp) for presentation, and the teachers were given a choice of times to attend. As well as an introduction to the study, the workshop included an explanation of stress management strategies and how they could be digitally supported. It was recognized that, although this study did not seek to identify sources of stress, some of which could be outside the workplace, COVID-19 was adding a new source of stress for many that would have to be acknowledged. Teachers were then presented with a rigorously compiled taxonomy, the design of which has been described elsewhere [46]. The taxonomy enabled teachers to have some limited choice regarding the technology they were to use for the study. These technologies were framed in the workshop as digital companions, a deliberate approach that sought to acknowledge the users’ autonomy [83]; emphasize the mediating, collaborative role of the technology [84]; and avoid any connotations of consumption. The set of consumer technologies offered is shown in Figure 1. After feedback forms were received, teachers were emailed to arrange payment of a flat participation fee to cover the costs of their chosen technology and schedule their first interview.

### Semistructured Interview Details

A total of 8 semistructured interviews were conducted by the first author with each teacher at around the summer half-term at a time convenient to the teacher and then again at the end of the summer term (Multimedia Appendix 1). In total, 75% (6/8) of the teachers took part in a further interview around the autumn half-term, having agreed to extend the original study to a time when they were back on school premises. Of the 25% (2/8) of teachers lost to follow-up, there was no response from one, and the other teacher cancelled her interview because of illness. Each interview sought to explore the teachers’ experiences of using their technology for managing their stress, what they valued, and what was significant. The interviews reflected the constructivist paradigm of the research, which sought to capture the teachers’ lived experience of the phenomenon of consumer technology use for stress management [85-87]. The interview questions followed the 5-phased structure outlined by Blandford et al [88]. The 3 semistructured interview scripts had themes to which contexts were applied: expectations in the first interview (early summer), adoption and experiences in the second interview (end of summer), and evolution of use on school premises in the third interview (autumn). However, each interview was also tailored to each participant based on, first, their technology choice and workshop feedback questionnaire and drew on the usability goals [89]*.* In the case of the second and third interviews, they were based on the teachers’ responses to the previous interviews and the surveys (see the following section). Methodological validity and integrity were achieved by applying high and relevant standards and several different strategies for data collection [86,90,91].

The questions sought to explore what using the technology and the data generated meant to the teachers as they sought to manage their stress. Each teacher’s responses were copied into a file and added to a pseudonymized case study folder in NVivo (version 12; QSR International) after the interview. The length of each interview ranged from 32 to 76 minutes.

### Open and Closed Survey Questions

The intended study spanned 6 to 7 weeks of the summer term, and survey links were sent by email soon after the halfway mark. In total, 3 sets of themed survey questions were sent during the data collection week to prompt thinking about their perception of the data, stress relief activities, and the influence of the work setting so far on their technology (Multimedia Appendix 1). The response rate was 100% (8/8) on the first and last days of the week and 75% (6/8) midweek. The answers informed the second and third interview questions. A total of 14 more closed questions were asked at the end of the interview in the extended study in the autumn to capture final thoughts on what had been significant and valuable or not from using the technology. These survey responses also provided more data for triangulation along with the workshop feedback forms and memos created during the interviews.

### Analysis Detail

All interviews were transcribed verbatim, with identifiers removed, and checked against the audio recordings for accuracy. Data from the interviews, surveys, memos, emails, and feedback forms were organized in NVivo using SimpleMind (versions 1.22 and 1.31; SimpleApps) to map the data, and codes were shared with the coauthors (AB and JEC). Notes and memos were made throughout the study and shared with the coauthors during analysis. Duration of technology use was based on self-report. The analysis was guided by a combined inductive (bottom-up from the data) and cognitive and social constructivist (the participants’ experiences and learning through interaction with others) thematic analysis. In total, 8 detailed descriptive case studies were compiled from the summer data, and 6 (75%) of these were extended to include data from the autumn term, adapting steps from 2 main sources [92,93]. A cross-case analysis was informed by interpretive phenomenological analysis to allow for exploration of meaning, patterns, and dynamics as well as idiographic accounts [94]. This approach enabled a deep analysis of the interrelation of the teachers’ roles, events, thinking, and responses within a particular context and the creation of narratives that acted as a patchwork of information from which similarities and differences could be identified (Multimedia Appendix 2 [86,95-97]). A summary of the teachers’ technology choices, years of teaching experience, chosen strategy and supported concept, rationale, duration of use, and reported use is shown in Table 1.

**Table 1:** Teachers’ technology choices for longitudinal study of stress management and summary of user experience.

Teacher number	Technology choice	Time as teacher (years)	Chosen stress management strategy and technology-supported concept	Rationale for choice	Duration of reported use (nighttime use indicated for the Withings smartwatch)	Reported stress management strategy or use
T1	Daylio and Wysa apps	>10	Cognitive and reflection; cognitive and self-management	Already used Fitbit. Apps looked easy to use, colorful, and with psychological and social relevance.	20 weeks continuously and then a few missed days before the autumn half-term	Used as planned plus self-management learning. Empathy applied to others in the last 6 weeks (autumn term)
T2	Withings watch—wearable	>10	Physiological and reflection or peer-to-peer social support	To understand sleep; quick to use and track activities	22 weeks (including nights)	Used as planned but unhelpful with sleep
T3	Withings watch—wearable	>20	Physiological and reflection or peer-to-peer social support	Wanted more data than Fitbit gave her and to understand sleep and make correlations between stress and activities.	22 weeks continuously (nighttime wear quickly discontinued)	Physiological reflection used; peer-to-peer social not used (as predicted by T3 at the start)
T4	Withings watch—wearable	<10	Physiological and reflection or peer-to-peer social support	Wanted to be able to track patterns in behavior and activity.	15 weeks continuously and then 4 times per week for the next 7 weeks (nighttime wear quickly discontinued)	Physiological and reflection; peer-to-peer social not used (T4 thought she would)
T5	Teacher Tapp app	>10	Educational and reflection	To learn and share with other teachers and access educational information	7 weeks; 2 days continuously and then occasional use for 7 weeks in the autumn term	Used as planned plus learning and empathy applied to others; added physiological and self-management
T6	Withings watch—wearable	>20	Physiological and reflection or peer-to-peer social support	Wanted something passive that could be reviewed for patterns between sleep, stress, exercise, and eating.	27 weeks continuously (including nights), included planned health leave for 6 weeks	Used as planned; peer-to-peer social not used when back in school (watch out of Bluetooth range of phone)
T7	Withings watch—wearable	>10	Physiological and reflection or peer-to-peer social support	Already used Teacher Tapp. Watch was passive, and T7 could review patterns between heart rate and stress.	7 weeks, intermittent wear	Occasional use of physiological reflection; peer-to-peer social not used; added occasional Teacher Tapp (education and reflection)
T8	Withings watch—wearable	<10	Physiological and reflection or peer-to-peer social support	Wanted to know how activity affects the body and pay more attention to physical demands	8 weeks continuously	Physiological reflection used; peer-to-peer social not used

## Results

### Overview

Of the 8 teachers interviewed twice in the summer, only 1 (12%) was working on the school premises, but by the autumn term, the 6 (100%) teachers involved in the extended study were all back on-site. The participants were ethnically diverse, but there was just 12% (1/8) of male teachers in both the summer and autumn studies (whereas men make up 35% of secondary school teachers). The youngest teacher was aged 28 years and the oldest was aged 58 years. COVID-19 hygiene rules meant that teachers were moving between classes in the autumn of 2020, year groups were segregated, and the school day was longer because of students’ staggered arrival and leaving times. All the teachers (8/8, 100%) had used technology for health or well-being before the study.

In total, 2 main themes were identified in both the summer and autumn term case studies, and 1 further theme was identified from the autumn analysis. First, in both school terms, the data collected by the wearables and contained in the apps powerfully demonstrated the physical and psychosocial demands of work and stressful situations. These data generated self-compassion (being kind and understanding toward oneself), prompting permission to destress, validating their subjective experiences, and increasing awareness of and generating empathy toward colleagues as well. Flowing from this permission, the second theme revealed that their stress management technology could also be a medium for indicating values. Using this technology enabled both a sense of belonging and being part of a community and could engender more trust through the autonomy it could support.

In the autumn term, the final theme built on the first 2 as teachers described how their understanding of their stress and value of self-care had, to an extent, become embedded and more intentional. They still interacted with their technologies but did not always need to before understanding that a situation was stressful and giving themselves permission to destress.

These themes are now described in more detail, with the teachers’ number, technology, and school term indicated.

### Theme 1: The Significance of Data Gave Teachers Permission to Self-Manage Their Stress

Teachers described how, having personalized their technologies, both the data and information on well-being and activity enabled reflection on the toll of teaching on them. This consciousness led to self-compassion and a realization that, as well as capturing the demand, the data could affirm the action taken to look after oneself.

#### Subtheme 1.1: Personalization Evolved Through Finding Goals and Boundaries

All teachers consciously curated the use of their technology over time according to their needs. They chose both their stress self-management strategies to be supported and the primary mechanism through which their technology could help them:

I was talking to one of my colleagues about it this morning...These apps prompt you to think differently and change your perspective and not go yes, I need to do that, or think about things at the end of the day, but actually reflect as you go through the day, and not just at the end of the day or the end of the week, etc...I guess what I’m doing is just adapting the app to think about you as a person because you can be very intentional of the things you put on there...

T1; Daylio and Wysa apps; summer term

In total, 67% (4/6) of the teachers who had the wearable used it in the summer term to set boundaries through the enabling of notifications, reminders, or alarms. Some used reminders to prompt breaks, eating and drinking, or exercising as, even though they had more autonomy and time to pause, the work-home boundary was blurred by working from home:

So, I’m constantly checking. I love just having a look at the app in the morning, just to see what the data is, and see what it is for the day. And then I can say, right, I need to do more of this today, or I need (to) try and do that a bit more.

T2; Withings smartwatch; summer term

Some teachers personalized their devices by selecting the information they paid attention to, for instance, heart rate or sleep data. In total, 50% (3/6) of the teachers who used the Withings smartwatch chose not to wear it at night, either because they did not want to know about their poor sleep as it made them more stressed or because, once good sleep had been affirmed, they no longer needed the confirmation.

In the autumn, 67% (4/6) of the teachers reported no further tailoring of their technology on top of what had already been undertaken to support stress management. The reasons included having already chosen what was meaningful (T1) and having had plenty of time to learn how to use the technology well during the lockdown (T4). A total of 33% (2/6) of the teachers reported being so tired by the autumn half-term that they sometimes forgot to use the app or put the watch on in the morning. One teacher described trying the food diary when back in school, although she discontinued the use of the meal planner because of the effort and finding it boring. Another teacher reported setting more alarms as she found that they were meaningful reminders to drink or take a breath.

#### Subtheme 1.2: Self-Compassion Led to Intrinsic Permission Sustained at the School Site

In the summer term, teachers who used the wearable reported that wearable collated data on sleep, calorie burn, heart rate, and activity gave them a better understanding of their bodies’ responses to work and stress throughout the day. Similarly, the teachers using the apps described taking time to reflect on emotional tracking, educational information on stress, or even questions related to current circumstances as heightening their awareness of the need to look after themselves. This understanding prompted self-compassion and seeing themselves as human beings, not just teachers. Combined with the knowledge of their stress management strategies, this implicitly gave each of them permission to look after themselves better:

The biggest thing about using the apps, is helping me to be self-conscious of me. Even like thinking how I’m running, how I’m doing things, how I’m sitting, how I’m walking...think I have more value for “me.” In fact, I’ve probably treated “me” a lot more harshly [in the past] it was never about me. It was about everybody else.

T1; Daylio and Wysa apps; summer term

All teachers (8/8, 100%) noted that their jobs were less physically demanding during the lockdown, when they were working from home, but the data still demonstrated the demands of teaching on the body. Making time for exercise had to become more intentional and was motivated by seeing their low-movement data. All those who used the wearable could see associations between feelings of stress and their heart rate, even when working from home. This underscored the experience of being human with bodies that reacted to stress and strain, not merely teachers doing a job:

The other day...I was trying to create a policy, basically, and just thinking about next year. I did my heart rate check and it was like, gosh clearly I’m stressed, it had gone up by like 30. Normally my heart rate is in the 60s, but it had gotten up into the 90s. I was like...I’m going to take a breather. I’m going to go for a walk and then come back and then reassess it. That was actually really interesting because I hadn’t, in the moment, I hadn’t really recognised that I was getting that stressed.

T8; Withings smartwatch; summer term

Only 12% (1/8) of the teachers did not report greater self-compassion as she had found being out of the school workplace setting a disincentive to using the wearable as she only wore a watch when going to work.

In total, 50% (3/6) of the teachers described in the autumn how the permission they had given themselves to look after their own well-being to manage stress continued in the school environment. One teacher testified that she now recognized her raised heart rate as an indicator of stressful situations and that this prompted her to take time to recover—an action of self-care that she had not previously had the information or insight to trigger:

And again, when I have seen my heart rate go higher or I know I’ve been really stressed, I’ve been trying, even if it’s just for five minutes, just to go and sit in my office and shut the door. Just for five minutes and just do something else or just sit there and just take a minute to just calm down.

T4; Withings smartwatch; autumn term

This reported self-compassion could then also lead to action, and the data generated enabled self-affirmation, as described in the next subtheme.

#### Subtheme 1.3: Self-Affirmation From Seeing Collated Data Contributed to Resilience

All teachers using the wearable in the summer term (6/8, 75%) described how being able to see their data constituted evidence of their efforts to manage experiences of stress, evidencing their worth, which can be termed *self-affirmation*. It affirmed when they had permitted themselves to relax, such as by walking or taking a time-out, or showed when efforts regarding nighttime habits had resulted in better sleep. Those using apps appreciated seeing their tracking or rewards:

I felt like the data was telling me that I’d worked hard enough, and I deserved it. So, I was like, well, I’ve done my bit and I’m just going to step out of it...Making sure I’m in bed and getting the hours that I need, which is quite good because it plots it for you. It says, it’s a sweet spot, you’ve had enough sleep.

T2; Withings smartwatch; summer term

Teachers still appreciated the patterns they could see in their data as proof of their self-care in the autumn, and one teacher described how the data had become a real-time prompt as well. Seeing the displays of self-care routines was meaningful in terms of understanding how they essentially contributed to resilience when in the thick of school activity:

So, in terms of it helping to remind me what I’m doing, what I need to do, it does in that way because it helps you to keep into a routine...So, if I’m not in routine and I don’t do certain things, like yoga, my Bible study...So, indirectly, it does [help with stress management] because it teaches you...It may not be in the moment say on Daylio...[but] it helps me to deal with day-to-day management of different things at school.

T1; Daylio app; autumn term

#### Subtheme 1.4: Confidence and Empathy Came Through Knowledge, Leading to Sharing

The heightened awareness of the impact of stress meant that all participants (8/8, 100%) commented on being more confident in sharing both the insights and the technology itself in discussions of stress, and this could generate empathy toward colleagues experiencing stress:

What I love about Wysa, it talks about remedies, and it gives you ideas, and it educates you on why people feel like that...I’m going honest because I know what triggers my stress...I think I’ve learnt more about other people.

T1; Daylio and Wysa apps; summer term

Back on the school premises, one teacher reported that her conversations regarding stress management had been with the students, not with fellow teachers. She had seen the relevance for herself in managing stress better through deliberately exercising more, and she also tried to demonstrate to the students the meditation feature that came with her wearable companion app. When this failed, she demonstrated Headspace (meditation app) instead and found that, despite having a challenging class, the students responded positively:

So, I thought I’d introduce it [the meditation on the watch] to my Year 10s, but I couldn’t do it with them, so I downloaded Headspace and I’ve used Headspace with my Year 10s. So, we meditate in form time and they love it. I’ve got the most difficult form group...it’s just quite interesting that [it gets] them calmer.

T3; Withings smartwatch; autumn term

### Theme 2: Technology Use Could Indicate Educator Values

#### Overview

Teachers identified that connecting through technology was important as it mitigated a sense of isolation. They described how it could also be a medium for indicating value by giving recognition of both autonomy and the need for them to be supported. This theme complements the first theme of permission to self-care that teachers described from having the technology and data.

#### Subtheme 2.1: Connectedness Through What Is Important to the Teacher

Teachers’ nuanced responses revealed the value of being able to choose to stay in touch, either with each other or with the wider educational context, or, conversely, not being disturbed. The Withings smartwatch messaging feature provided the ability to stay connected with family or colleagues when working from home, which reduced the stress of potentially missing out. This gained extra salience when teaching from home, when the watch became a “fail-safe”:

So I have three devices set up when I teach. I have my laptop with that [an electronic pen] attached to it now, to one side of me I have my iPad [for the chat function] and then I have my phone on as well, [so] that I can see what they can see...that’s where the watch comes into its own. Because then, anything that comes through, comes onto my watch.

T6; Withings smartwatch; summer term

Teacher Tapp alerts were described as helping frame the school day (going off at 3:30 PM, a reminder of the end of lessons), appreciated particularly during lockdown and working from home, and giving the sense of connection with other teachers, an unanticipated benefit during lockdown:

But over this period, I’ve used it (Teacher Tapp) a bit more, because I want to see what people are saying about Covid and stuff...I thought, great this is fantastic...it’s nice to see what people are saying or what people are thinking during this pandemic.

T7; Withings smartwatch and Teacher Tapp app; summer term

Experiences of connectedness varied more in the school environment in the autumn, but being able to see family messages was mentioned by some as reassuring:

So...without having to have my phone out...I could see the content of the message, and I knew it wasn’t something that was urgent, but I knew I was informed of what was going on...so in that way, yes, I have been using it in school.

T2; Withings smartwatch; autumn term

#### Subtheme 2.2: Trust Through Autonomy and Being Valued

Teachers described different levels of trust between themselves, other staff, and the school’s leadership, with 75% (6/8) of the teachers expressing reticence on being open about stress—sharing insights would depend on trusted relationships. With leadership, a climate of trust was associated with both the sense of autonomy given to teachers and how value is shown, for instance, through well-being support. This included the provision of respite in the workplace from the frontline, be it in the form of protected breaks, opportunities for camaraderie, or off-duty privacy such as a staff room. A couple of teachers mentioned how leadership offering a digital tool for stress self-management in continuing professional development could facilitate autonomy:

You’re assigned a CPD [continuing professional development] and you have to go...While for this (using Teacher Tapp) it would be up to you to engage. You know, do you want to fill out the questionnaires, do you want to do the reading? So there’s some autonomy to what’s taking place, and you as the individual have control about the success in your outcomes.

T5; Teacher Tapp app; summer term

One teacher described that stress was perceived as a reason for teachers leaving the profession. As a head of year, she often felt that she did not know how to help fellow staff, but she could now recommend technology for personal stress management:

I am quite interested in teacher retention...and think that stress is one of the big causes for drop out...We don’t really talk about stress...people frequently tell me they are stressed but I don’t necessarily feel that I’ve got many...strategies to help them...(Now) I might say, this is a tool that’s helped me. Yes. Definitely. I think I would probably be quite honest about it and be like, oh yes, it’s really helped me managing this stress.

T8; Withings smartwatch; summer term

Without exception, teachers felt that more effort to appreciate their work and stress had been made by the leadership during the pandemic. The leadership had demonstrated this by asking about family circumstances, reducing teaching demands, enabling web-based chats or worry-box emails, and providing well-being tips and more line management support that most described as continuing in the autumn term.

A further finding was derived from the analysis of the autumn data, which signified an embedding of the learning described in the previous themes.

### Theme 3: Learned Helpfulness Meant Reduced Reliance on Their Technology

Teachers returned to school in the autumn having used their technology for at least 12 weeks, including over the summer holidays. The frequency of technology use dropped in the autumn term, with lack of time and opportunity given as reasons, but most teachers revealed how lessons learned could be applied in the moment.

They described being able to apply learning from the use of their technology and reflecting on the data, although some commented that working from home had given them the opportunity to learn and practice changes in habits, which may not have happened had their use begun on the school premises:

I think things that I learnt over the lockdown, and with the technology, I definitely have...tried to actively act on those lessons as much as I can. I think it’s probably come from more a place of learning from the tech previously, as opposed to using it at the moment. I’m not sure how I would have used it if I’d have just turned it on at the start of term. I think it was interesting to have those two experiences, actually.

T4; Withings smartwatch; autumn term

Learned helpfulness (where action or insight has become embedded and the original source of help is no longer required) did not always translate into behavior. One teacher described how her desire to look after herself in school was overridden when in the physical setting, surrounded by occupational demands. To still benefit, she would have to plan to use the technology more deliberately. She reflected that she could be more proactive, setting reminders to take a moment for herself, have lunch, or simply have a moment’s rest:

I think maybe what I might do is set myself a few little alarms as well, daytime alarms, which are triggers for me to go look at my timetable and actually set a time, even if it is to eat...and just take ten minutes or 15 minutes out...Maybe I need to be more organised and be more aware of my wellbeing and what’s important for me. And use the data and use the technology more to effectively manage my day.

T2; Withings smartwatch; autumn term

## Discussion

### Overview

The duration of this longitudinal study of 8 teachers’ experiences of stress management using consumer technologies was from 6 to 27 weeks, and for 75% (6/8) of the participants, it extended from the end of summer to the autumn half-term. This presented the opportunity for a long period of exploration.

**Figure 2:**
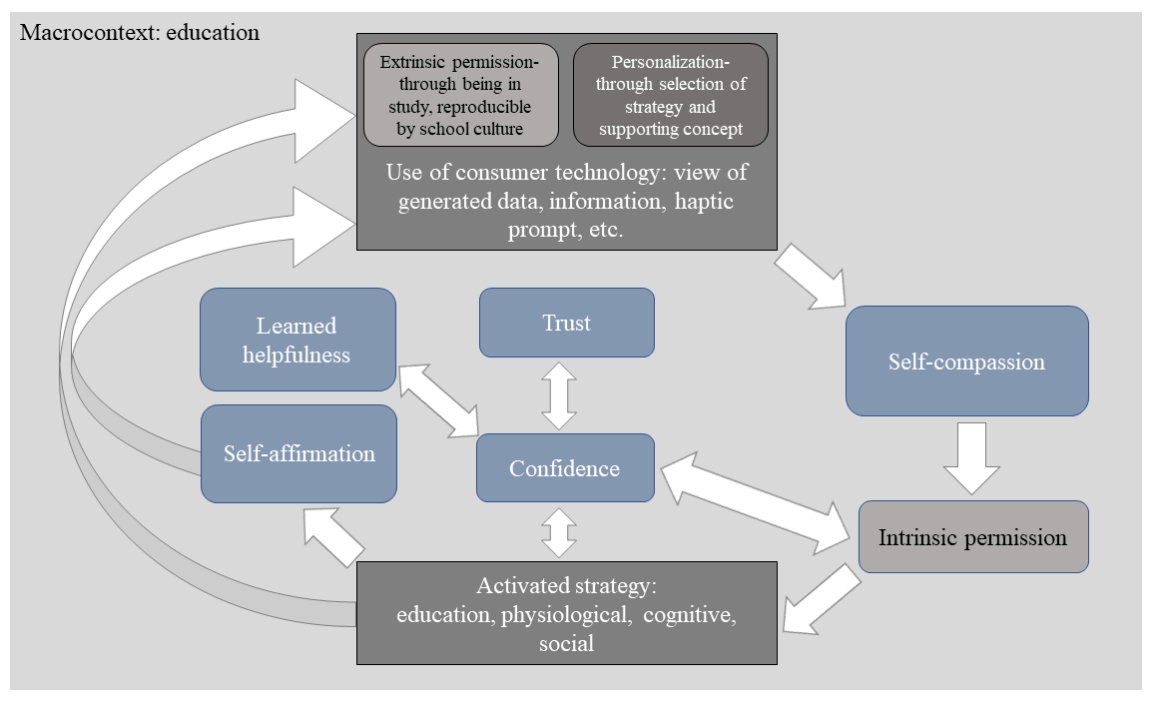
Cycle of experience with the technology described in interviews, where meaningful data were generated leading to self-compassion and intrinsic permission to self-manage stress and continue use.

The themes generated from the cross-case analysis indicate an overarching virtuous cycle of experience initiated by extrinsic permission and the personal choice of technology. Interacting with the technology generated meaningful data, the existential significance of which permitted self-compassion and empathy, prompting confidence and self-management strategies, the value of which stimulated further engagement (Figure 2).

Together, these findings imply that consumer technologies can play a valuable role in teachers’ stress management, which we will now explore in more detail.

### Qualification in Context

Data meaningfully captured some of the stress of teachers’ roles, and permitted compassion and self-care to resonate with themes of real-world awareness [76] and permission, which has been reported as a mechanism that facilitates teacher well-being in a taught (nondigital) mindfulness study [98]. Similarly, self-compassion has been found to improve through digitally supported stress management among health care students, where they were educated about the physiological impact of stress and provided with supported relaxation via an app [99]. This study validates this finding and demonstrates the explanatory power of contextually meaningful data alone, particularly real-time heart rate data. Such data are humanized by the context [100], and they gain existential significance [65]. When data quantified the toll of teaching through physical demand or capturing teachers’ emotional variations, this deeper, macrocontextual “educational” understanding of stress on themselves generated self-compassion. A few mixed feelings were expressed on awareness of stress at the personal, micro level, aligning with previous work highlighting the risk that perception of stress can, for some, lead to increased stress [101-103]. Given that we know the importance of stress management for good mental and physical health, this suggests that, when raising awareness of stress, it would be prudent to always offer signposting to or supply stress management interventions. Similarly, for some teachers in the study, choosing not to wear the Withings smartwatch at night was motivated by not wanting to know about poor sleep. Future research could also look at whether there are other sensor data that could capture the demands of work that teachers or other public-sector workers could find meaningful.

Data also took on the deeper significance beyond quantification to achievement, affirming teachers’ use of self-management strategies, creating affective ties [55] similar to the “qualified-self” (Boam and Webb, 2014, quoted by Lupton [104]). In this case, self-affirmation from thinking through rather than just from data was supported by visualizations enabling deeper reflection on achievements [55,105]. This complements and extends previous research on the positive framing of data in PI [106] and shows that reflection as a component of tracking can come after, not simply before, acting [107]. It also adds to the findings that self-affirmation through cognitive reappraisal using given self-affirmation statements for teachers generates positive emotions [108].

### Enabling Choices for Motivation and Meaning

Through the populated taxonomy [46], teachers were given the *choice* of technology for stress management and how this could support their self-care strategies. In accordance with self-determination theory (SDT) [109], this autonomy is a key motivator leading to engagement and, therefore, one possible explanation for the lack of attrition in this study, with no one abandoning their technology before their final interview. Autonomy has been described as a key component *within* design architecture for engagement [110]. These findings extend the engagement narrative to *without* design, indicating that contextually appropriate strategies and concepts should guide users’ choice of technology. This reflects the understanding that there is no one-size-fits-all stress intervention [76] and the hypothesized personal preferences such as time, location, and personal relevance (ie, external autonomous factors) are important [111].

These preferences are linked to the finding of personalization. Personalization through curating frequency, naming, and timing of messages and haptic alerts was important, and all were directed toward personally meaningful activities or boundaries. The Daylio app allowed for some refinement to tracking, and teachers made conceptual links between, for instance, heart rate changes on their wearable companion app and the day’s events. Personalization (also called tailoring and customization) is a well-attested facilitator of engagement with apps [112] and a suggested guideline in PI stress management practices [66]. This study’s findings confirm the value of an individual approach to considering what data are relevant given peoples’ contexts [66,113].

### Relatedness in the School Context

The themes also affirm the value of social connectedness, noted in previous digital health research as highly valued [114] and important for engagement in broader digital mental health studies [115]. For some teachers, this study affirmed the concept of relatedness mediating thriving through technology design, as posited by the Motivation, Engagement, and Thriving in User Experience model, which draws on SDT [116]. Seeing both other teachers’ responses on Teacher Tapp and messages from colleagues and family generated a sense of connection and belonging. This insight emphasizes the importance of occupational and social connection at times of separation, which will be worth remembering should teachers ever have to work remotely again. Owing to COVID-19 restrictions, this study was not able to verify the mutual support for activity through shared data previously shown with wearables offering social connection [117], including among teachers [40]. Digital connectedness with colleagues was less important when back on the school premises, where the shared environment permitted social (mesocontextual) conditions for relatedness even with COVID-19 restrictions on staff movement.

Teachers’ confidence in understanding stress was demonstrated through compassion toward colleagues. Mesolevel (eg, culture) and microcontextual (eg, personal)-level perceptions of trust and support are predictors of lower stress and burnout among teachers [7]. Personal confidence was generated because the data were contextually situated and interpreted. Of note were the teachers who used the Teacher Tapp and Wysa apps. The participant who chose Wysa found it empathetic, as designed [58], but also that its use generated empathy for others, a transferability that appears to be novel, although hinted at when a gamified, visually responsive avatar was used in a web-based stress self-help system [75]. This finding also complements our other findings on relatedness. The 25% (2/8) of participants who accessed Teacher Tapp during the lockdown appreciated knowing how other teachers were feeling in uniquely isolated conditions. The digitally enabled outcome of confidence can be seen to be mediated by both connectedness and competence, which are components of SDT.

The final theme corroborates the conclusions of other studies that a reduction in the use of technology does not necessarily equate to abandonment [106]. Resilience without reliance on activity tracking should be considered a strength of PI, and it is important not to expect ongoing dependency on technology [118,119].

As a counter to the “learned helplessness” by Seligman [120], 83% (5/6) of the teachers in the autumn described an embedded notion of “learned helpfulness” as they were able to apply stress management strategies back in school without using technology in the moment. This does not contradict the initial need for sustained engagement [121,122], but it does raise questions about the duration of the effect and designing in the opportunity to reminisce [106]. This study benefited from a long extension that allowed for the study of evolving meaning making and demonstrated some embedding of positive habits, which has been shown in other shorter studies on stress management in the workplace [76].

### Strengths and Limitations

To our knowledge, this is the first qualitative study of high school head of year experiences with technology-enabled stress self-management. Rich, contextualized data from situated experiences and evolution of use were collected over an extended period, enabling deep and detailed insight into their existential significance. A total of 8 teachers still completed the summer study despite the pressures of COVID-19. This longitudinal study provided evidence of the value of technology to teachers for stress management support and the value of digitally supported self-care in an occupation renowned for its stressfulness.

A potential weakness is that participation meant that there was some extrinsic motivation and would have raised awareness of stress and self-care, so a digital placebo effect is a possibility but possibly less of a concern given our focus on experience rather than efficacy [123-125]. However, this placebo effect could have resulted in more positive bias toward the intervention, which would have to be addressed in future studies on efficacy. In addition, as only half of the 16 teachers who were approached before the pandemic took part and 2 were lost to follow-up in the extended period, volunteer (selection) bias needs to be taken into account in the findings. In addition, only 12% (1/8) of the participants were male, which could have skewed the findings. Most participants had time to adopt their technology in a quieter environment as most worked from home at the start of the pandemic, even though their working days could still be long and contain different sources of stress than usual. Further research could explore the experiences of technology adoption while in school under usual circumstances. Another limitation was the restricted choice of consumer technologies. A limited choice was given so that assurances could be given on suitability, availability, usability, security, validity, and cost. Thus, there was a trade-off between assurance and full autonomy, something that could be explored further. In addition, apart from Wysa, the teachers did not choose technologies that offered treatment and guided self-management or were entertainment based. This could be due to the small number of participants; a preference for the immediacy of a wearable; or, in the case of treatment, not wanting to see stress as something to be medicalized, as one teacher commented. This too could be further investigated, as could examining the incorporation of technology-supported stress management into teacher training with an active control (against non–technology-supported stress interventions).

### Conclusions

Stress remains a significant problem for most teachers, including those in leadership roles. This research is one of the first to critically explore heads of year’s experiences of using consumer technology to support their stress-coping strategies. These findings highlight the importance of taking a situated approach to understanding the real-world, holistic significance and value of data. This should encourage similar studies to refine digital interventions for all teachers and other workers in high-stress environments. The implications of the analysis are that school leaders should encourage consumer technology use to generate a deeper, school-wide understanding of stress and that such use can inform how stress can be mitigated.

The findings of this study are context and condition specific yet indicate that designers would benefit from considering context-based interactions in occupational environments for different health conditions. In collaboration, developers, employees, and policy makers could all better ensure that consumer technologies can improve workforce health outcomes. Achieving this would require going beyond user acceptance testing to capturing user experiences in the real world.

## Appendix

Multimedia Appendix 1 Interview guide and survey questions.

Multimedia Appendix 2 Details of cross-case analysis.

## Abbreviations

iCBT: internet-based cognitive behavioral therapy
PI: personal informatics
RQ: research question
SDT: self-determination theory

## Data Availability

The data sets generated and analyzed during this study are available from the corresponding author upon reasonable request.
