# Supplementary material for: High School Teachers’ Experiences of Consumer Technologies for Stress Management During the COVID-19 Pandemic: Qualitative Study
Source: JMIR Form Res. 2023 Nov 15;7:e50460. doi: 10.2196/50460 (PMC10687684; doi:10.2196/50460)

# Appendix

# Longitudinal Study Semi-Structured Interview Guide 1 (Summer - entry)

**Introduction to cover**

- **Thanks:** for their time. Remind them of the Education Support Partnership helpline contact details on the information sheet if they feel they want to talk further or receive support after this interview.
- **Topic Reminder:** This interview is an introduction to the ‘practical’ study where they will be using their DCT(s) in their workplace – wherever that may be – and its potential to support teachers manage their stress.
- **Confidentiality**: The name of the participants will only be known by the researchers and not be revealed to anyone. The participants will be identified by an ID and all potentially identifying information will be removed. Individual teachers and those from different schools are being interviewed and findings aggregated, so no answers can be tracked back to a particular school.
- **Participation:** Do not share information you don’t want to, there are no right or wrong answers, it is about their views – but please be as honest as they can.
- **Recording:** the session will be zoom-audio-recorded, transcribed and analysed by researchers using NVIVO software, one of the most used widely software for qualitative analysis.

**Interview Framework**

1. Please could you describe a typical working day as it is now?
2. Please could you describe a typical working day when you are in school?

**Individual - Background**

*(Informed by Workshop Feedback Form)*

- Technology Choice:
- Reason for choice:

Based on the tech you have chosen, the stress management strategies that it supports fall into the category of…

1. Is this a stress-management strategy that you currently use?
2. Have you used any digital tools to support your stress management before now?
3. How do you think you will use the DCT?
4. What are your hopes or expectations from using it? Both in terms of benefits and drawbacks?
5. Do you think time or effort will be an issue?
6. Three frequently mentioned symptoms in my first study were dehydration, isolation, insomnia – do any of these resonate with you?

**Physical – environmental context**

*(Informed by Workshop Feedback Form)* Where do you think you would use the DCT?

1. Do you feel more or less physically isolated now you are working mainly from home compared to when in school? More / Less / Both / Same
2. Can you briefly say how or why?

*From Ball (Educational contexts – Situated, material)*

- situated contexts (such as locale, school histories, intakes and settings)
- material contexts (for example, staffing, budget, buildings, technology and infrastructure)

*From HCI Perspectives*

- Workspace
- Conditions
- Layout

1. How do you think the type of school that you are in impacts the way that you experience stress? (Given they are WFH, compare workspace, conditions, layout between home and school)
2. Are there particular aspects of stress that you think the DCT will help you manage? (see feedback comments)

**Social – relationships context**

*(Informed by Workshop Feedback Form)*

1. Has your school Principal communicated about supporting teachers’ work-related stress in the past? Y/N
2. Has the Corona virus made it easier for you to talk about work stress? Y/ N / Same
3. Are you more or less able to manage your work stress when working from home?

More / Less / Both / Same

1. In your feedback you said (about discussing stress) that ….
2. Do you think your school colleagues will influence your use of the DCT? HOW
3. Do you think you will mind them knowing that the DCT is to help you manage stress?

**Organisational – Culture and Control contexts**

*(Informed by Workshop Feedback Form)*

1. Prior to March 20th did your school leadership encourage collaboration between staff? Y / N

Has this changed since March 20^th^? Y / N

1. Would you describe your school as:

- Having a positive learning environment? Y / N / Don’t know
- Fostering positive relations with the community Y / N / Don’t know
- Including teachers in decisions related to school policy? Y / N / Don’t know

Obviously at the moment you are working from home, so the next question should be considered from two perspectives

1. Firstly, when working from home, in the course of your working day, is there anything you can anticipate that will stop you using the DCT – regulations, disruptions, workload, routine? If so, what are they?
2. And secondly, when working on the school premises, is there anything you can anticipate that will stop you using the DCT – regulations, disruptions, workload, routine? If so, what are they?

**Next steps**

**Study Duration**

June 1^st^ – w/c July 13th

**Half-way survey**

The survey will take place during the week of June22nd-26^th^

A reminder will be sent the week before

**Keeping in touch**

Please feel free to contact me with questions at any time.

I am not requesting that you keep a diary of your technology use, but any notes or comments you want to make as you go along would be fine – or simply send them in a text to me 07973312358 or email me at [j.manning.17@ucl.ac.uk](mailto:j.manning.17@ucl.ac.uk) and I can collate them.

**Thank you**

Finally – thank you once again for being prepared to give up your time and take part in this study. I very much hope that you will find the experience valuable and interesting.

#

# Longitudinal Study Semi-Structured Interview Guide 2 (Summer – Intended Exit)

**Introduction notes as above**

**Questionnaire Framework**

Covid19

1. How many days have you been in school since half term?
2. What do you think has been the impact of Covid19 on your experience of work stress?
3. How have the changes to your routine changed your use of technology for a) work b) yourself?

Tailored questions for DC choice

You Chose (DC Name) which enables (Technology concepts)

To help with your (as appropriate) stress management strategies (SMS) (relaxation, exercise, seeking social support etc)

1. Were there any issues with learning, functionality, amending or remembering how to use the DC?
2. Do you think that overall as a result of having the DC/data there has been a change in: (A/B)

| **Action** | **In School** | **Covid (Work from Home)** | **Details** |
| --- | --- | --- | --- |
| *Prompt* | Effectiveness – awareness enough? | | Why (motivation), did novelty wear off, |
| Exercise |  |  |  |
| Relaxation |  |  |  |
| Social connections |  |  |  |
| Sleep |  |  |  |
| Drinking (water/ tea!) |  |  |  |
| Other action as a result of the data |  |  |  |

1. Did you feel that the data related to your stress levels – that it gave you a valid correlation?
2. What encouraged you to use the DC (facilitators)
3. What discouraged you to use the DC? (drawbacks/ challenges/ barriers)

Tailored questions based on previous interview

(N.B. Questions follow that relate to expectations from Entry Interview)

1. Comparisons with …
2. What aspect most useful – reminders/ alarms/ steps/ calories etc
3. You mentioned chatting to colleagues online and via an app – which one do you use?
4. Was your heart rate interesting to monitor?
5. Was time or effort would an issue?
6. Has there been any change to your quality or quantity of sleep?

Withings Watch (example)

(if not previously covered)

Stress

1. The watch provided data on which to reflect – it also enabled meditation, social support, setting alerts and reminders. What did you find the most helpful to looking after yourself?
2. Did you think the watch was particularly useful to you as a teacher/ Head of Year in managing stress, and if so, why?
3. Has using the Watch made you think that managing your stress is more important than you did before?

User

1. What would be your goals if you carried on using the watch?
2. There was roughly a 50:50 split on those who had mentioned their DCT stress management to colleagues; Would you recommend a fellow teacher buying one?
3. What more could be done to help participants engage with occupational digital mental health?

HCI & Contexts


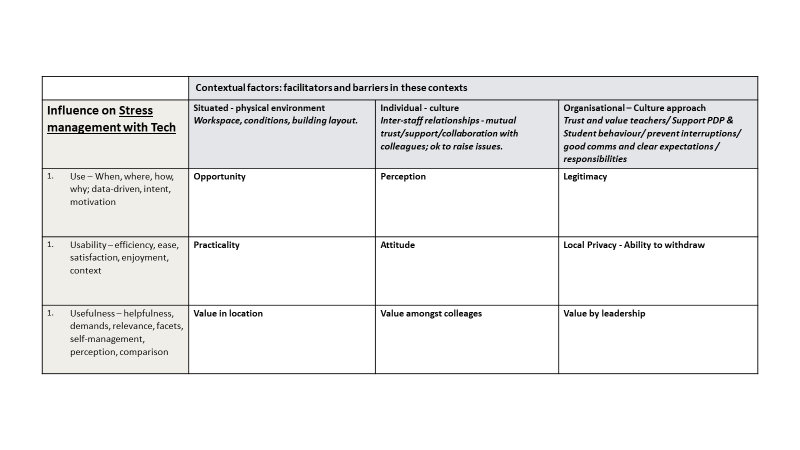
*This section is to investigate the influences of your job role, location and colleagues on your stress management as supported by the watch. The table below will be populated as viewed on shared screen by the participant and interviewer. The participant will be asked to consider the influence of the context on stress management of the use, usability and usefulness of the technology as a support for their stress management strategies*

Final Q

1. What would the ideal digital companion be like to support your stress management in your role as a Head of Year?

# Longitudinal Study Semi-Structured Interview Guide 3 (Autumn – Extended Exit)

**Introduction notes as before**

**Questionnaire Framework**

Exemplar for Withings Watch

The stress-management strategy that you identified was the following:

1. Educational – such increasing awareness of the stress response
2. **Physiological – such as relaxation or exercise**
3. Cognitive – such as problem solving or time management or
4. Social – such as seeking social support or social skill development

The technology you chose to try out utilises the technique of

1. **Fostering reflection by making health and contextual information available**
2. Suggesting treatment adjustments or activities, and guided self-management
3. Peer-to-peer social support
4. Utilizing entertainment

General questions

1. You have just had the first full half-term with pupils back in school since the start of the pandemic – what have been the similarities and differences compared to pre-pandemic?
2. Following on from above (to prompt more detail if need be)
   1. Physical environment
   2. Culture – individual / relational
   3. Culture - Organisation
3. What have been your particular experiences of stress during this half term?
4. Now you are back in the school workplace, what has your experience been of using your watch/app to help with your stress management? (Differences between now and summer term).
5. If we consider a stressful moment in school – whatever that happens to be – do you think the technology you chose helped you in the moment (at the time of the stressful incident)?
   1. If yes, what is it about the technology that helps?
   2. If no, what is it about the technology that doesn’t help?
6. If we consider a stressful moment in school – whatever that happens to be – do you think the technology you chose helps you withstand the pressure (or in building resilience - more able to withstand or adapt to stress)?
   1. If yes, what is it about the technology that helps?
   2. If no, what is it about the technology that doesn’t help?
7. Thinking in more detail about the barriers and facilitators to the tech helping you, we’re going to think about these again from the physical, social and cultural perspectives that we thought about in July.


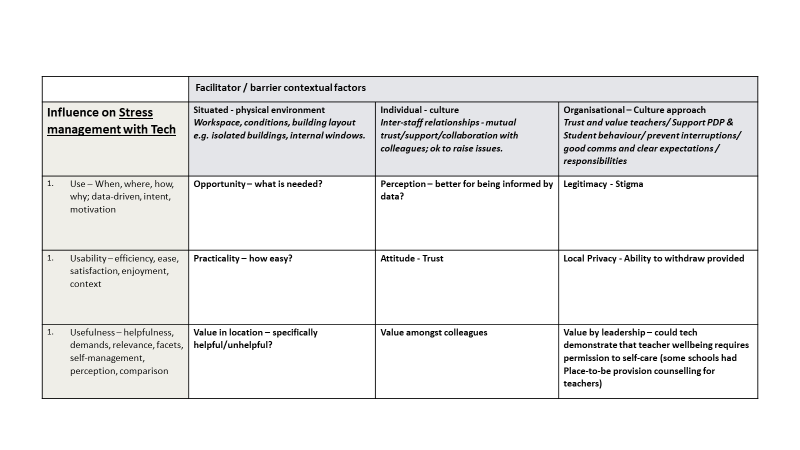


1. In previous interviews, teachers indicated that they liked seeing the data the technology collected or portrayed for a variety of reasons. Would you agree or disagree with the following statements on having the data:
   1. The data confirmed your stress-management strategy Y/N
   2. The data affirmed the importance of your self-care Y/N
   3. The data allowed you to frame and plan your day Y/N
   4. The data allowed you more opportunity to reflect on your wellbeing Y/N
   5. The data was accessible in school Y/N
   6. The ability to act on the data was dictated by the circumstances you were in Y/N
   7. The data influenced the amount of activity or exercise you did Y/N
   8. The data prompted better time management Y/N
   9. The data affirmed your hard work and validity of having a break Y/N
   10. The data in-the-moment indicated that you were more stressed that you realised Y/N
   11. The data helped you to feel connected to your peers Y/N
   12. The data helped you to feel less isolated - no feels isolated from close colleagues Y/N
   13. The data was helpful for improving your sleep
   14. The data was helpful for drinking or eating more regularly Y/N
   15. The data helped you understand and manage your colleagues stress Y/N
2. Now having used the technology during the summer and autumn terms, how would you improve it?
3. If there was one thing you would like to change in school that would help you to self-manage stress what would it be?
4. Do you think the same change would be helpful to your colleagues, or is there another change that would make a difference for the majority of your colleagues in managing their stress?

**Tailored questions (which may have been covered in responses above)**

1. You spoke in July about finding your voice and how this had reduced your sense of stress – has this continued do you think?
2. Do you think the watch has helped you more with managing your own stress or dealing with your stressed colleagues?
3. Are you still looking at your sleep patterns?
4. Have you carried on trying to be more active and drinking more? (was she more active in the summer as planned?)
5. You mentioned your heart rate rising due to other people’s behaviour or management expectations of you doing things in a certain way – does that still happen or have you found a way to deal with that?!
6. You liked having all the notifications on your watch – did you continue to appreciate these (and not having to have you phone with you?)
7. Have you felt more or less isolated from colleagues? Or less able to avoid interruptions from kids?
8. Has the SLT management been more supportive this term?
9. Have you tried going to a different toilet this term?!

Keeping in touch

Please feel free to contact me with questions at any time.

**Thank you**

Finally – thank you once again for being prepared to give up your time and take part in this study. I very much appreciate all the time you have given to this study.

# Longitudinal Study Open and Closed Question Survey Responses (Summer Term)

Survey questions in week 4 of longitudinal study on Monday, Wednesday and Friday (NB One response from the Friday was captured during the second interview)


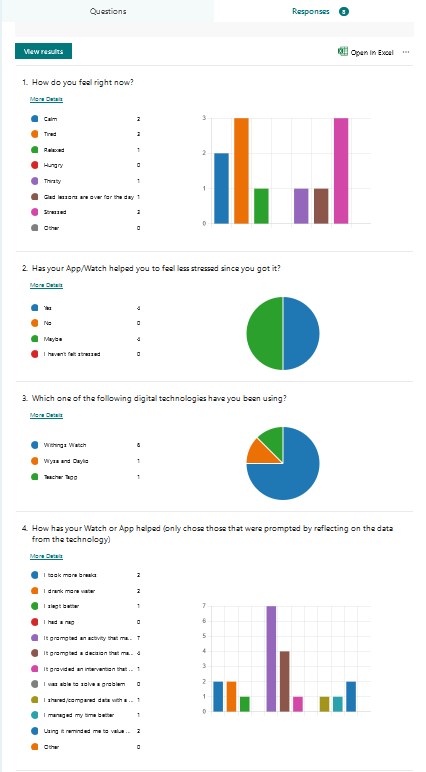


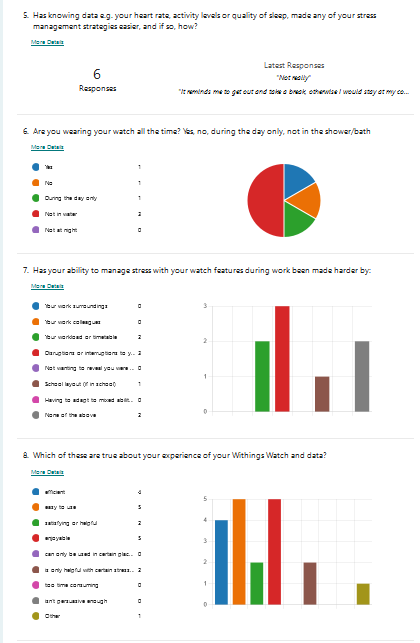


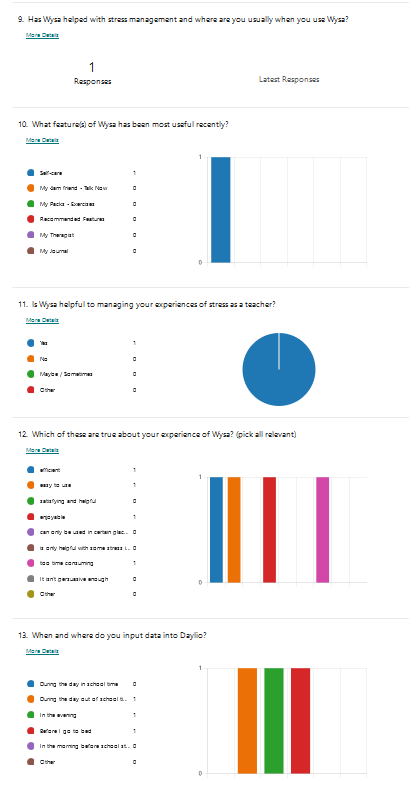


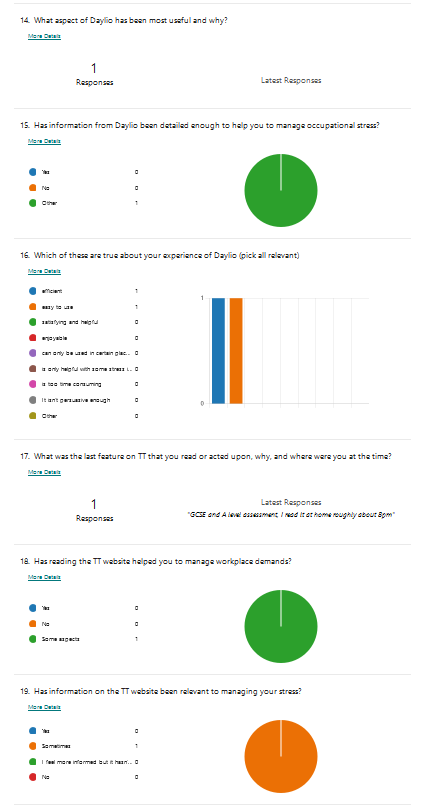


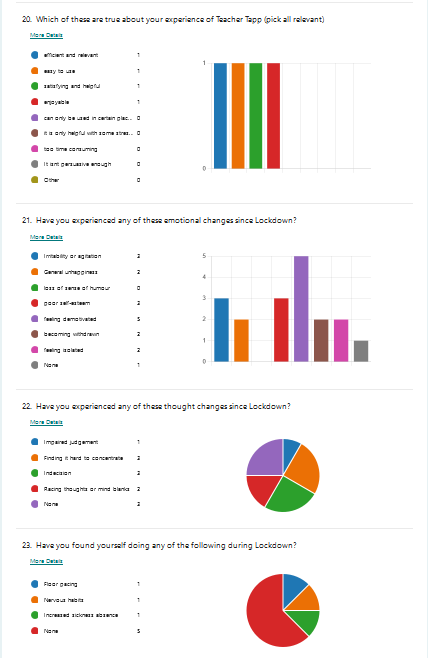


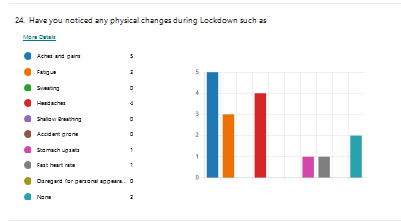


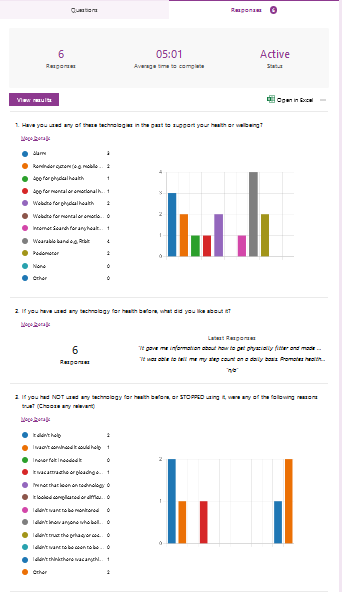


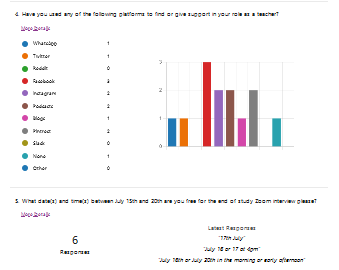


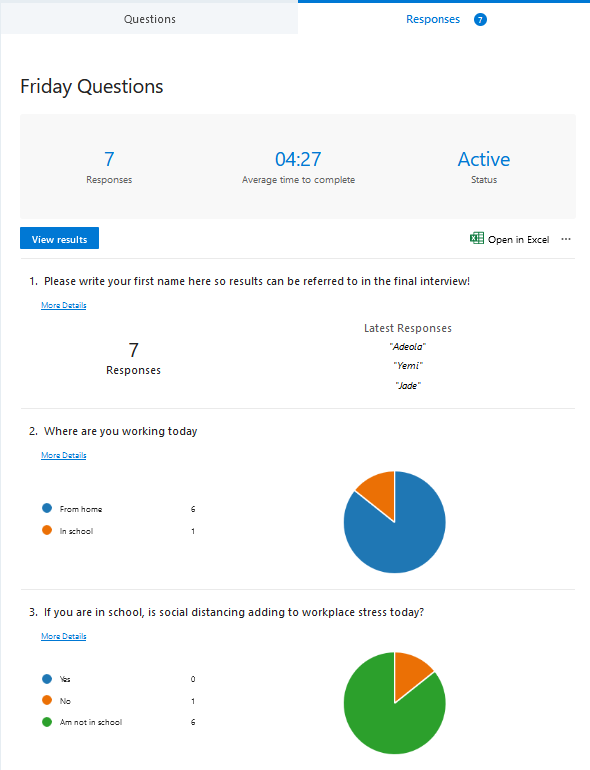


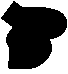


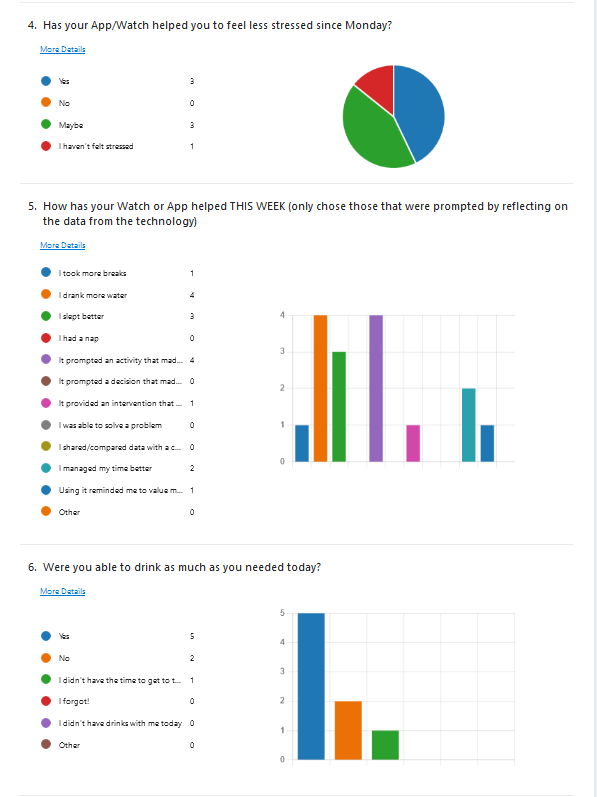


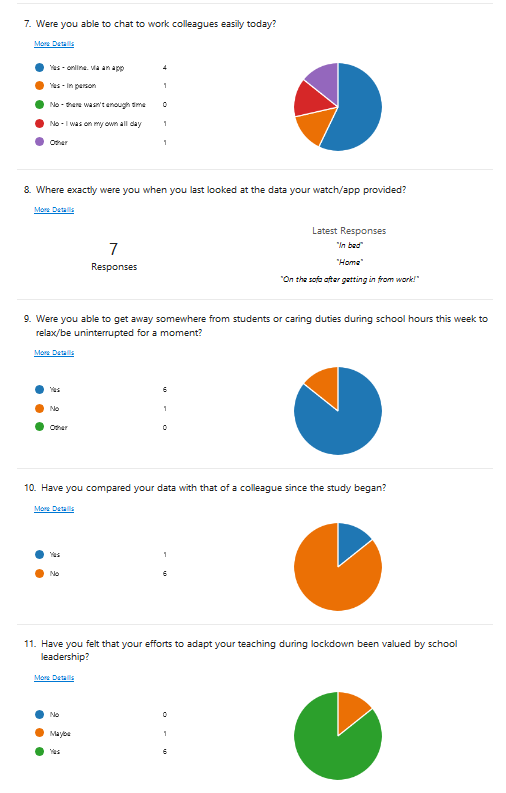


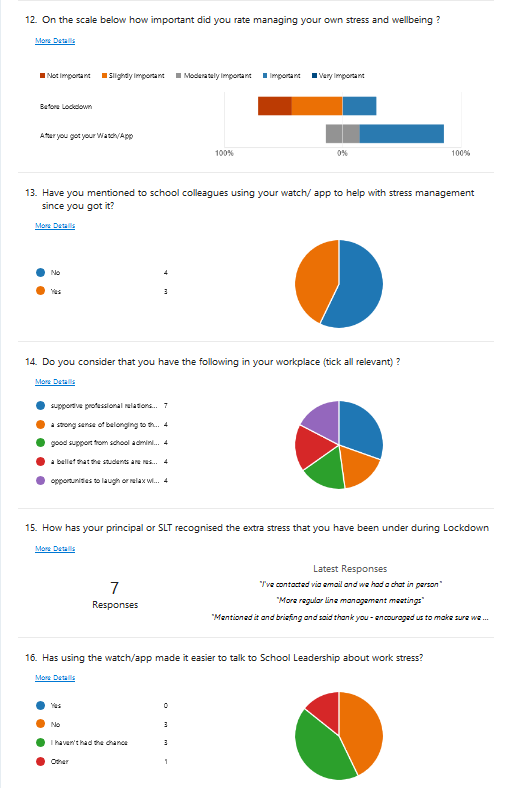


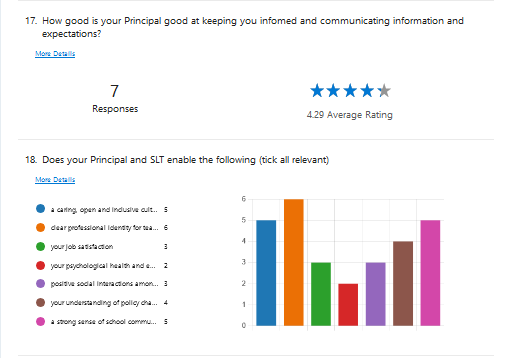

Supplement: Multimedia Appendix 1 [file formative_v7i1e50460_app1.docx]
