# Supplementary material for: High School Teachers’ Experiences of Consumer Technologies for Stress Management During the COVID-19 Pandemic: Qualitative Study
Source: JMIR Form Res. 2023 Nov 15;7:e50460. doi: 10.2196/50460 (PMC10687684; doi:10.2196/50460)
Supplement: Multimedia Appendix 2 [file formative_v7i1e50460_app2.docx]

Appendix 2

The steps for analysis were adapted from two main sources. Firstly, the creation of descriptive case studies as described by Yin [92]. Secondly, a within-case and cross-case phenomenological approach illustrated by Kavanaugh from the three different approaches to such analysis described by Ayres, Kavanaugh, & Knafl [93], enabled a systematic approach to the analysis. Creating case-studies allowed the development of detailed profiles for each teacher from the data. This provided an understanding of subtle differences in their experiences and appreciate how these were shaped by context (Powell & Bodur [96]). These within-case analyses enabled immersion in the data from each teacher and gain a sense of their experiences of adoption and use of their technology (Miles & Huberman [97]).

Summary of Strategy for case-studies and cross-case analysis

| Strategy | Purpose | Analytic focus | Product |
| --- | --- | --- | --- |
| Analytic immersion in all interviews | Familiarisation with the data | Within all cases | Coding and recoding categories |
| Analytic immersion in all case data | Triangulation of the data | Within each case | Annotated case files |
| Immersion in each case file | Identify important aspects of the phenomenon: adoption, evolution of and influences on use | Within each case | Case studies describing the experience of the phenomenon with key quotations |
| Comparison of key quotations | Identify similarities and differences between the cases | Across cases | Experiences common to all participants and where they diverge; nascent themes |
| Reconnection of key quotations to interviews | Ascertain fidelity to original accounts | Within and across cases | Verification and refining of themes |
| Free writing | Reflection and to begin narrative | Within and across cases | Answering the RQ on adoption, use, evolution in the contexts |
| Review narrative | Explanatory power of themes and statements | Themes | Final narrative |
